# Gonadotropins and Their Association with the Risk of Prediabetes and Type 2 Diabetes in Middle-Aged Postmenopausal Women

**DOI:** 10.1155/2019/2384069

**Published:** 2019-08-05

**Authors:** Anna Stefanska, Paulina Cembrowska, Justyna Kubacka, Magdalena Kuligowska –Prusinska, Grazyna Sypniewska

**Affiliations:** Department of Laboratory Medicine, Collegium Medicum, Nicolaus Copernicus University, Sklodowskiej-Curie 9, 85-094 Bydgoszcz, Poland

## Abstract

Recent studies have suggested that a low concentration of follicle-stimulating hormone (FSH) is associated with a higher prevalence of metabolic disturbances in postmenopausal women. In this study, we aim to evaluate the association between FSH, luteinizing hormone (LH), and LH/FSH ratio values and the risk of insulin resistance (HOMA-IR >2.0), prediabetes (IFG), and type 2 diabetes in a 5-year prospective study in postmenopausal women. 114 postmenopausal women were divided into 4 groups: group 1 (baseline and follow-up normoglycemic women), group 2 (normoglycemic women at baseline progressing to IFG), group 3 (women with baseline and follow-up IFG), and group 4 (women with baseline IFG progressing to diabetes). Baseline and follow-up anthropometric measurements and blood collections were performed. Serum/plasma was assayed for glucose, HDL-C, TG, C-reactive protein (CRP), 17beta-estradiol, estrone, insulin, thyroid-stimulating hormone (TSH), FSH, and LH. Homeostatic model assessment of insulin resistance (HOMA-IR) and LH/FSH ratios were calculated. The baseline concentrations of FSH and LH statistically decreased across all four groups (the highest concentrations in group 1 and the lowest in group 4; *p* < 0.001). A logistic regression analysis showed that a 1 SD decrease in the *z*-score of FSH concentration is associated with a threefold increased risk of IFG and a fivefold increased risk of HOMA-IR of >2.0 and diabetes. The LH concentration had odds ratio (OR) values about two times lower than the FSH concentration. The ORs of the LH/FSH ratio were only significant for IFG. In conclusion, FSH concentration is strongly associated with insulin resistance, prediabetes, and diabetes in postmenopausal women with normal or impaired fasting glucose. LH and the LH/FSH ratio are also related to metabolic disturbances after menopause, yet to a lesser extent.

## 1. Introduction

Dysregulation of gonadotropin (LH and FSH) secretion is related to abnormalities at the hypothalamic-pituitary axis, infertility, and metabolic disturbances in premenopausal women and men. It was observed that an elevated LH/FSH ratio is associated with obesity, insulin resistance, and an increased risk of type 2 diabetes in women with polycystic ovary syndrome (PCOS) [1, 2]. A recent study from the Study of Women's Health Across the Nation (SWAN) has shown that a higher risk of developing diabetes was associated with a slower rate of FSH increase during early perimenopause [3]. The data on the relationship of gonadotropins to metabolic disturbances and diabetes in postmenopausal women are scarce and mostly focused on FSH [4–7]. Recently, two prospective studies have shown an inverse association between FSH concentration and the risk of diabetes [8, 9]. Yet, there are no prospective studies estimating the association of FSH, LH, and LH/FSH with metabolic disturbances in postmenopausal women. For this reason, we aim to evaluate the association between FSH, LH, and LH/FSH ratio values and insulin resistance, prediabetes, and diabetes in a 5-year prospective study in Polish postmenopausal women.

## 2. Materials and Methods

### 2.1. Participants

At baseline, women were recruited from the total group of 270 postmenopausal middle-aged women without diabetes who participated in the Menopause and Metabolic Syndrome Study in the Department of Laboratory Medicine of the Nicolaus Copernicus University in Bydgoszcz between 2007 and 2010. Between 2012 and 2015, all the women were contacted by phone and/or mail and invited to participate in the follow-up evaluation. Finally, 122 women agreed and signed written informed consent forms (the Bioethics Committee at the Nicolaus Copernicus University in Torun, Collegium Medicum, in Bydgoszcz). Reasons for nonparticipation among those who were lost to follow-up were as follows: distance from the study center, loss of interest, loss of contact, change of residence, and other health problems. The baseline characteristics of the women who did not participate in the follow-up are shown in Table 1.

Eight women with IFG at baseline were excluded because they reverted to normoglycemia.

At baseline, the women were selected according to inclusion and exclusion criteria. The inclusion criteria were as follows: at least 1 year after the final menstrual period (FMP) and aged between 45 and 60 years, while the exclusion criteria covered clinical evidence of diabetes mellitus type 1 and/or 2; liver and/or thyroid disorders; acute inflammation; surgical menopause; premature menopause; a history of PCOS; a serum CRP > 10 mg/L; a TSH concentration below 0.35 or above 4.94 *μ*IU/mL; and the taking of medications such as insulin, oral antidiabetic agents, hormonal replacement agents, and thyroid and/or antithyroid agents.

114 women were divided into 4 groups, according to the diagnostic criteria of prediabetes and diabetes:

*Group 1*. NFG to NFG: normoglycemic women at baseline and after 5 years of follow-up

*Group 2*. NFG to IFG: normoglycemic women at baseline progressing to prediabetes

*Group 3*. IFG to IFG: women with baseline and follow-up prediabetes

*Group 4*. IFG to diabetes: women with baseline prediabetes progressing to diabetes

86 women with baseline homeostatic model assessment of insulin resistance (HOMA-IR) of <2.0 (selected from the group of 114 women) were additionally divided into 2 groups according to values of HOMA-IR after 5 years of follow-up:

*Group 1:* women with baseline and follow-up HOMA-IR < 2.0 (*n* = 48; 91% women from the group NFG to NFG and 9% from the group NFG to IFG)

*Group 2:* women with baseline HOMA-IR < 2.0 progressing to HOMA-IR > 2.0 (*n* = 38; 53% of women from the group IFG to IFG and IFG to diabetes, 37% from the group NFG to IFG, and 10% from the group NFG to NFG)

This division aims to estimate the association between the baseline concentration of gonadotropins and insulin resistance defined according to the HOMA-IR values.

### 2.2. Diagnostic Criteria

Type 2 diabetes was diagnosed according to a fasting glucose level in plasma ≥7.0 (≥126 mg/dL) repeated on two consecutive days or self-reported, physician-diagnosed diabetes and the use of glucose-lowering medications for diabetes. Impaired fasting glucose (IFG; prediabetes) was indicated by a fasting glucose level in plasma of 5.6-6.9 mmol/L (100–125 mg/dL) and a normal fasting glucose (NFG) of <5.6 mmo/L (100 mg/d) [10]. An insulin-resistant state was diagnosed in women with HOMA-IR > 2.0 [11].

### 2.3. Baseline and Follow-Up Measurements

Height, weight, waist circumference (WC), and systolic and diastolic blood pressure were measured using standard methods. Occurrences of disease, physical activity, current smoking habits, and medication use were determined by a questionnaire developed during a visit at the Department of Laboratory Medicine. 10 women with self-reported diabetes were treated with glucose-lowering agents (metformin and sulfonylureas).

Baseline and follow-up blood were collected in the early morning (7.00–9.00 am) after an overnight fast (12 h). Serum was assayed for HDL-C, TG, and TSH, and plasma was measured for glucose on an ARCHITECT ci8200 (Abbott Diagnostics). Serum FSH and LH levels were measured on an AxSYM (Abbott Diagnostics) and the serum 17beta-estradiol level (E2) was measured on an Elecsys 1010/2010 (Roche Diagnostics). CRP was measured using a BN II System nephelometer (Siemens Healthcare Diagnostics, IL, USA). Serum estrone levels and insulin concentrations were determined using the sandwich ELISA method (Estrone DRG MEDTEK, intra-assay precision 6.7%-9.1%, inter-assay precision 6.9%-11.7%; insulin: DRG MEDTEK, intra-assay precision 2.8%-4.0%, inter-assay precision 2.6%-3.6%). HOMA-IR value was calculated by dividing the fasting insulin concentration (mU/L) and the glucose concentration (mmol/L) by 22.5 [11]. Follow-up EDTA blood samples were analyzed for glycated hemoglobin (HbA1c) using a Bio-Rad VARIANT II turbo (HPLC).

### 2.4. Statistical Analysis

The data were expressed as means (± standard deviation SD) or medians (25th and 75th percentile), respectively, for the Gaussian and non-Gaussian distribution. The Shapiro-Wilk test was applied to test the Gaussianity. The two independent groups were compared using the Student or Mann–Whitney *U* tests. The two dependent groups were compared using the *T* test or Wilcoxon rank sum test. The comparison of more than two groups was made using ANOVA or Kruskal-Wallis tests. To perform an analysis of covariance and multiple regression, variables with non-Gaussian distribution were natural log-transformed. Partial correlation coefficients were calculated for baseline values of the parameters with adjustment for WC. Pearson's chi-squared test was used for categorical variables. To estimate prediabetes, diabetes, and the insulin-resistant state odds model, a multivariate logistic regression, based on variables transformed to *z*-scores, was performed. Standardized values (per 1 SD increase/decrease) were used for comparability between the odds ratios for FSH and LH and LH/FSH ratio. We assessed the standardized values by calculating *z*-scores (according to mean and standard deviation of the study population). The significance of coefficients in the logistics models was tested using Wald chi-squared statistics. In all logistics models, FSH, LH, and LH/FSH ratio were included (base model 1). Additional models were adjusted for age, WC, BMI, CRP, TG, HDL, systolic blood pressure, and 17beta-estradiol. All covariates were related to the occurrence of prediabetes and diabetes. The quality of fit for each logistics model was assessed by the Hosmer-Lemeshow chi-squared test. Statistical significance in all analyses was accepted at the level of 0.05 or below (STATISTICA 13, StatSoft).

## 3. Results

The baseline characteristics of women lost to follow-up and of those participating is presented in Table 1. We observed that women who did not participate in the follow-up had significantly higher BMI but a similar baseline prevalence of IFG and HOMA-IR > 2.0.

The baseline and follow-up characteristics of the women is shown in Table 2. All baseline groups were matched for age and years after menopause. Baseline groups had similar concentrations of TSH and estrone. Statistically different values of BMI, WC, glucose, insulin, HOMA-IR, HDL-C, TG, and CRP were observed. The baseline concentrations of FSH and LH statistically decreased across all four groups, even after adjusting for WC (the highest concentrations in group 1a and the lowest in group 4a; *p* < 0.001). We observed statistically significant lower concentrations of FSH and LH in normoglycemic women who progressed to prediabetes and women with prediabetes who developed diabetes, in comparison to women who remained normoglycemic or prediabetic (FSH; *p* < 0.001, and LH; *p* < 0.01). The values of the LH/FSH ratio slightly increased, but this trend was not statistically significant (*p* = 0.09). All women had baseline values of LH/FSH below 1.0. The lowest concentrations of 17beta-estradiol were observed in group 1a, and the highest in group 4a (*p* = 0.04), but we did not find statistically significant differences between groups 1a and 2a (*p* = 0.89) or 3a and 4a (*p* = 0.96). FSH and LH concentrations suffered a statistically significant decrease in all groups after 5 years of follow-up (1a versus 1b: FSH, *p* < 0.01, LH, *p* < 0.001; 2a versus 2b, *p* < 0.05; 3a versus 3b, *p* < 0.001; 4a versus 4b: FSH, *p* < 0.05, LH, *p* < 0.01). The values of LH/FSH also decreased after 5 years of follow-up, yet to a lesser extent (Table 2). Women with follow-up HOMA-IR < 2.0 (group a) had significantly higher baseline FSH and LH concentrations in comparison to women with follow-up HOMA-IR > 2.0 (group b) [94.6 (71.9-115.9) versus 61.0 (51.6-78.5), *p* < 0.001, for FSH and 35.0 (32.4-47.8) versus 26.9 (23.2-34.6), *p* < 0.001, for LH]. The median values of the LH/FSH ratio were higher in women with follow-up HOMA-IR > 2.0 (group b) [0.41 (0.33-0.49) versus 0.43 (0.37-0.59), *p* = 0.04] (data not shown).

FSH and LH concentrations were strongly and negatively correlated with BMI (*r* = −0.61 and *r* = −0.47; *p* < 0.001, respectively) and WC (*r* = −0.66 and *r* = −0.53; *p* < 0.001, respectively). For that reason, all correlations with biochemical parameters and blood pressure were adjusted for WC. FSH is significantly and negatively correlated with values of glucose (*r* = −0.29, *p* < 0.001), HOMA-IR (*r* = −0.21, *p* < 0.05), TG (*r* = −0.18, *p* < 0.05), and CRP (*r* = −0.29, *p* < 0.001). LH is negatively correlated with values of insulin (*r* = −0.19, *p* < 0.05) and HOMA-IR (*r* = −0.20, *p* < 0.05). LH/FSH did not correlate significantly with the parameters of carbohydrate and lipid metabolism. LH/FSH ratio only correlated with CRP (*r* = −0.33, *p* < 0.001) (data not shown).

The logistic regression analysis showed significant associations of FSH with the 5-year odds of developing IFG, diabetes, and HOMA-IR > 2.0 in the crude model as well as in models adjusted for all covariates (Table 3). A 1 SD decrease in the *z*-score of FSH concentration was associated with a threefold increased risk of IFG, a fivefold increased risk of HOMA-IR > 2.0 and diabetes in models adjusted for WC (*p* < 0.01). The relationship of LH with the 5-year odds of developing prediabetes was not significant. However, LH was significantly associated with the 5-year odds of developing diabetes (*p* < 0.05; except in the models adjusted for WC and BMI) and an insulin-resistant state (HOMA-IR > 2.0; *p* < 0.05). The increase in the 5-year odds of developing diabetes and an insulin-resistant state, when the value of the marker concentration decreased by 1 SD, was about two times higher for the models based on FSH than for the models including LH. A 1 SD increase in the *z*-score of LH/FSH was only associated with prediabetes (*p* < 0.05; except in the model adjusted for CRP), but this association was weaker in comparison to the models based on FSH (Table 3).

## 4. Discussion

Prediabetes and type 2 diabetes are major public health problems, especially in postmenopausal women. Several studies indicate that about 25%-35% of the white population with IFG develop diabetes over a follow-up period of 5 to 6 years, and this percentage increases in subsequent years [12, 13]. It seems that a need exists to identify additional potential biomarkers that may be helpful in assessing the risk of developing IFG and diabetes.

### 4.1. The Association of FSH with Metabolic Disturbances after Menopause

Recently, several studies have demonstrated a negative association between FSH concentration and obesity, features of metabolic syndrome, an increased risk of diabetes, and atherosclerotic burden in postmenopausal women [4–7, 14].

In this study, we evaluated the association between values of FSH, LH, LH/FSH ratio and insulin resistance, prediabetes, and diabetes in a 5-year prospective study in postmenopausal women. To our knowledge, this is the first prospective study comparing concentrations of the two principal gonadotropins and gonadotropin ratios in metabolic disturbances after menopause. The results of our logistic regression analysis suggest that FSH concentration is most strongly associated with insulin resistance, prediabetes, and diabetes in comparison to values of LH and LH/FSH ratio. In 2001, Malacara et al. observed lower FSH concentrations in postmenopausal obese women; they concluded that lower FSH is related to higher estrogen exposure through a mechanism unrelated to insulin resistance [15]. Numerous studies have confirmed a strong negative relationship between obesity and FSH concentrations, which was also observed in this study [4–7, 14]. It was also found that weight loss leads to a slight increase in FSH among overweight postmenopausal women [16].

Our two earlier studies showed that postmenopausal FSH concentration is associated with metabolic syndrome in Polish women [4, 5]. Wang et al. observed that postmenopausal women with a concentration of FSH in the lowest quartile had threefold higher odds of developing diabetes in comparison to women with FSH levels in the highest quartile [7]. Bertone-Johnson et al. established the cutoff point of 50 IU/L as the point above which postmenopausal women aged 53-73 had a lower prevalence of diabetes [9]. A Finnish study found that high postmenopausal FSH levels were associated with a lower atherosclerotic burden, independent of estradiol, adiposity, and other factors [14].

In this study, we also found that FSH concentration is most strongly associated with insulin resistance and prediabetes in postmenopausal women. Similarly, Wang et al. observed that the risk of prediabetes occurrence decreases across FSH quartiles [7].

### 4.2. The Potential Mechanisms Responsible for the Relationship between FSH and Metabolic Disturbances

Higher estrogen synthesis in obese postmenopausal women may be associated with the aromatization of androgens to estrogens in the adipose tissue, which results in the inhibition of FSH secretion in negative feedback. In our study, similar to other studies, it was shown that an inverse relation between diabetes and FSH concentration is independent of obesity [7, 8]. Moreover, previous research suggests that a negative correlation between FSH and metabolic features and diabetes persisted after adjustment for E2 [5, 7]. In the present study, we found that E2 and estrone concentrations did not differ statistically between groups 1a and 2a and 3a and 4a, while FSH concentrations were markedly different in these comparisons. These results may suggest that a negative association between FSH concentration and metabolic disturbances is not completely accounted for by obesity and estrogen exposure, and other mechanisms must be taken into account to explain this relation. One of the mechanisms may be associated with the regulation of FSH concentration by activins and follistatin. Activins stimulate FSH secretion from the pituitary gland, and follistatin is a protein that suppresses this secretion by the neutralization of activins. Reame et al. observed that a menopausal increase in FSH concentrations is regulated by the sustained presence of activin A with age-dependent reductions in follistatin [17]. Follistatin and activins may be involved in the regulation of insulin sensitivity and inflammation status (activin A protects against hyperglycemia, hyperinsulinemia, and inflammation) [18–20]. It was also suggested that follistatin may be one of the adipokines produced by white adipose tissue [21]. Thus, this evidence may lead to the hypothesis that the follistatin-activin axis may play a role in the regulation of insulin sensitivity or inflammation after menopause, when hypothalamic-pituitary-gonadal negative feedback is limited. Another mechanism may be related to the more sialylated and less sulfonated structure of FSH after menopause. This change in structure is associated with FSH having a longer half-life, which may be negatively associated with obesity [22]. In an animal study, Renault et al. observed that the simultaneous administration of FSH and LH enhances the insulin response to glucose load in female dogs [23]. Krysiak et al. evaluated the effect of metformin on serum gonadotropin levels in postmenopausal women; they observed that high-dose metformin treatment reduced the concentration of FSH, and these effects correlated with an improvement in insulin sensitivity in diabetic women [24]. Other authors showed a positive relation between FSH concentration and sex hormone binding globulin expression (low SHGB is a well-known risk factor for diabetes) in postmenopausal women [25]. The nongonadal function of FSH is possible due to the presence of extragonadal FSH receptors, e.g., in adipose tissue, bones, hepatocytes, and blood vessels [26]. It was found that FSH has an angiogenesis effect in some tumors and promotes lipid biosynthesis in the adipose tissue [27, 28].

### 4.3. The Association of LH/FSH Ratio with Metabolic Disturbances after Menopause

An elevated LH/FSH ratio is considered to be an indicator of metabolic disturbances in premenopausal women with PCOS, although not all authors confirm this hypothesis [29]. In this study, the results of the logistic analysis do not indicate a significant association between the values of LH/FSH ratio and the occurrence of diabetes and insulin resistance in postmenopausal women. We found only a weak association between LH/FSH ratio and prediabetes. The correlation analysis only showed a significant relation between LH/FSH ratio and CRP. So far, two cross-sectional studies have evaluated the association between LH/FSH ratio and metabolic disturbances in postmenopausal women, outside the context of the PCOS diagnosis. Beydoun et al. observed that obesity, insulin resistance, blood pressure, triglycerides, and metabolic syndrome were not associated with LH/FSH ratio. They observed that CRP concentration is positively associated with an LH/FSH of >1.0, and an LH/FSH of >2.0 negatively correlates with glucose levels in postmenopausal women aged 35-60 [30]. In contrast, Zhao et al. showed that LH/FSH ratio was significantly associated with visceral obesity and lipid accumulation product. However, they did not observe significant differences in glucose and HOMA-IR values across quartiles of the LH/FSH ratio in Chinese women over 55 years old [31]. Thus, the results are contradictory and do not indicate the diagnostic usefulness of LH/FSH ratio in predicting diabetes in postmenopausal women. These differences in results may be partially caused by the decrease in the values of LH/FSH ratio with advancing age. Beydoun et al. were able to analyze the values of LH/FSH ratio above the designated cutoff points of 1 and 2 because the women in their study were younger [30]. In this study, all women had values of LH/FSH ratio below 1.0. For such low values, there are no fixed cutoff points with a documented diagnostic significance.

### 4.4. The Present Study Has Certain Limitations

The most important limitation is the small size of the study groups, which may reduce the potential of this study. For that reason, we could not perform multifactor adjustments of our analyses. The results of this study should be verified on a larger sample size. In this study, the response rate to a follow-up survey was 45% (270/122). The low response rate decreased the statistical power of this study. However, women who were lost to follow-up had a similar baseline prevalence of IFG and HOMA-IR > 2.0 in comparison to respondents, which may reduce the impact of this loss. Another limitation of our study is the fact that diabetes was only diagnosed by FPG, without HbA1C and 2-hour plasma glucose.

## 5. Conclusion

This prospective study suggests that FSH concentration is significantly associated with insulin resistance, prediabetes, and diabetes in postmenopausal women with normal or impaired fasting glucose. LH and the LH/FSH ratio are also related to metabolic disturbances after menopause, yet to a lesser extent.

## Figures and Tables

**Table 1:** Baseline characteristics of women lost to follow-up and participating in the follow-up.

	Women lost to follow-up (*n* = 148)	Women participating in follow-up (*n* = 122)	*p*
Age (years)^a^	54.3 ± 4.5	54.1 ± 4.1	0.69
BMI^a^	29.3 ± 5.8	28.2 ± 4.5	0.009
WC (cm)^a^	92.3 ± 13.4	90.4 ± 11.9	0.20
Years after menopause^a^	5.5 ± 4.0	5.6 ± 4.2	0.80
Glucose (mmol/L)^a^	5.42 ± 0.56	5.39 ± 0.67	0.68
HOMA-IR^b^	1.52 (1.0–2.4)	1.48 (1.0–2.3)	0.8
HOMA-IR > 2.0 (%)	32	35	0.65
Prevalence of IFG (%)	36	35	0.8
HDL (mmol/L)^b^	1.53 (1.32–1.73)	1.6 (1.34–1.84)	0.07
TG (mmol/L)^b^	1.22 (0.98 − 1.67)	1.28 (0.97–1.74)	0.40
CRP (mg/L)^b^	1.46 (0.66–3.36)	1.22 (0.57–2.34)	0.06
FSH (mIU/mL)^b^	68.7 (54.9–94.8)	74.8 (56.3–98.6)	0.20
LH (mIU/mL)^b^	30.9 (23.1–40.2)	32.9 (24.4–42.8)	0.31
Systolic blood pressure (mmHg)^b^	128.0 (116.0–138.0)	130.0 (114.0–140.0)	0.40
Diastolic blood pressure (mmHg)^b^	80.0 (76.0–87.0)	81.0 (78.0–89.0)	0.80

^a^Data expressed as means (±SD); ^b^Data expressed as medians (25th and 75th percentiles).

**Table 2:** Baseline and follow-up characteristics of the groups.

Parameters	Group 1a baseline (NFG to NFG) (*n* = 54)	Group 2a baseline (NFG to IFG) (*n* = 20)	Group 3a baseline (IFG to IFG) (*n* = 26)	Group 4a baseline (IFG to diabetes) (*n* = 14)	*p* for trend baseline groups	Group 1b follow-up (NFG to NFG) (*n* = 54)	Group 2b follow-up (NFG to IFG) (*n* = 20)	Group 3b follow-up (IFG to IFG) (*n* = 26)	Group 4b follow-up (IFG to diabetes) (*n* = 14)	*p* for trend follow-upgroups
Age (years)^a^	53.4 ± 3.0	53.8 ± 5.0	54.7 ± 5.0	56.1 ± 2.0	0.13	58.8 ± 3.2^#^	59.0 ± 5.7^#^	61.0 ± 4.5^#^	61.3 ± 2.0^#^	0.08
BMI^a^	26.1 ± 3.7	28.4 ± 4.0∗	28.9 ± 2.7	34.7 ± 3.8!	<0.001	26.7 ± 2.7	29.3 ± 3.8!	30.3 ± 2.5^$^	34.3 ± 3.1!	<0.001
WC (cm)^a^	84.6 ± 9.9	89.6 ± 9.8	92.3 ± 7.0	108.4 ± 8.7!	<0.001	85.0 ± 7.8	94.4 ± 11.4!	96.2 ± 7.0^$^	104.6 ± 5.5!	<0.001
Years after menopause^a^	5.2 ± 3.6	5.8 ± 4.4	6.0 ± 5.1	5.0 ± 2.4	0.6	10.4 ± 3.9^#^	10.1 ± 4.4^#^	11.2 ± 5.4^#^	10.3 ± 2.1^#^	0.46
Glucose (mmol/L)^a^	4.92 ± 0.3	5.16 ± 0.34∗	6.07 ± 0.34	6.36 ± 0.46∗	<0.001	5.05 ± 0.28^#^	5.82 ± 0.25^!,$^	6.0 ± 0.48	6.99 ± 1.1^&,@^	<0.001
Insulin (mIU/mL)^b^	5.6 (4.6-7.0)	7.0 (4.2-8.9)∗	9.1 (6.1-12.2)	9.5 (5.5-17.2)	0.002	5.7 (3.3-8.6)	10.5 (3.5-13,5)^!,@^	9.8 (8.2-11.9)	16.7 (9.1-29.4)^!,#^	<0.001
HOMA-IR^b^	1.27 (0.97-1.47)	1.66 (1.0-1.88)	2.49 (1.61-3.37)	2.33 (1.50-4.80)∗	<0.001	1.35 (0.73-1.95)	2.37 (0.79-3.14)^!,$^	2.61 (2.32-3.1)	5.8 (2.7-9.1)^!,#^	<0.001
HOMA-IR > 2.0 (%)	7.4	20.0	66.7	57.0	<0.001	19.2	55.6	91.7	100.0	<0.001
HbA1c (mmol/mol)^b^	—	—	—	—	—	34.0 (31.0-38.0)	38.0 (37.0-39.0)	39.0 (37.0-41.0)	41.0 (39.0-44.0)∗	<0.001
HDL (mmol/L)^b^	1.74 (1.55-1.97)	1.39 (1.19-1.79)^&^	1.45 (1.30-1.63)	1.35 (1.27-1.84)	<0.001	1.58 (1.37-1.79)^#^	1.35 (1.14-2.02)∗	1.45 (1.22-1.58)	1.37 (1.22-1.50)^$^	0.002
TG (mmol/L)^b^	1.14 (0.90-1.16)	1.02 (0.80-1.75)	1.59 (1.20-2.02)	1.94 (1.48-2.38)	<0.001	1.13 (0.89-1.44)	1.27 (0.89-1.45)∗	1.41 (1.22-1.58)	1.24 (1.16-1.55)^$^	0.002
CRP (mg/L)^b^	0.70 (0.30-1.30)	1.25 (0.52-5.70)^!^	2.14 (1.43-3.14)	3.50 (1.44-4.90)^!^	<0.001	0.63 (0.42-2.22)^$^	1.55 (0.96-2.28)^!^	2.20 (1.69-2.78)	3.67 (1.28-5.30)∗	<0.001
TSH (*μ*IU/mL)^b^	1.07 (0.73-2.02)	1.30 (1.20-2.20)	1.14 (0.56-2.13)	2.03 (1.90-2.49)^&^	0.06	1.13 (0.79-2.01)	1.67 (0.79-2.09)	1.09 (0.80-1.93)	1.66 (1.27-1.90)	0.39
17beta-estradiol (pg/mL)^b^	10.1 (5.0-24.1)	12.1 (4.9-26.0)	19.0 (14.0-34.0)	22.0 (11.0-29.0)	0.04	11.4 (4.9-22.0)	20.5 (18.0-38.0)∗^,$^	11.9 (4.9-16.0)	5.9 (4.9-8.3)∗^,#^	0.03
Estrone (pg/mL)^b^	43.2 (38.7-49.6)	35.9 (32.1-67.8)	41.9 (39.2-53.4)	40.7 (35.6-70.0)	0.3	49.8 (41.2-70.5)^#^	42.8 (44.3-59.9)^$^	50.6 (43.1-63.2)^@^	49.4 (46.2-53.8)	0.59
FSH (mIU/mL)^b^	94.3 (73.4-110.9)	67.6 (57.6-78.3)^!^	63.0 (56.0-78.5)	44.3 (38.0-55.34)^!^	<0.001	78.5 (61.2-111.0)^$^	62.3 (53.0-69.1)∗^,@^	51.8 (47.2-69.2)^#^	39.9 (36.0-46.8)∗^,@^	<0.001
LH (mIU/mL)^b^	36.4 (31.2-47.3)	34.2 (29.7-35.2)^&^	29.2 (23.3-35.0)	24.2 (16.3-30.8)^&^	<0.001	28.0 (23.3-38.6)^#^	28.1 (26.6-34.2)^@^	22.6 (20.1-27.1)^#^	17.8 (14.9-25.4)∗^,$^	0.002
LH/FSH^b^	0.40 (0.32-0.48)	0.46 (0.42-0.57)∗	0.46 (0.38-0.54)	0.52 (0.41-0.58)	0.09	0.37 (0.29-0.44)^@^	0.45 (0.41-0.55)∗	0.39 (0.34-0.49)^$^	0.40 (0.38-0.56)	0.17
Systolic blood pressure (mmHg)^b^	123.0 (104.0-131.0)	127.0 (124.0-130.0)	130.0 (125.0-146.0)	132.0 (130.0-150.0)^&^	0.05	120.0 (114.0-123.0)^&^	131.0 (122.0-138.0)	132.0 (126.0-140)	130.0 (114.0-140.0)	0.009
Diastolic blood pressure (mmHg)^b^	80.0 (73.0-89.0)	82.5 (85.0-86.0)	80.0 (78.0-89.0)	81.0 (80.0-92.0)	0.47	80.0 (77.0-84.0)	86.0 (76.0 - 99.0)^&^	89.0 (76.0-90.0)	86.0 (71.0–90.0)	0.04
Lipid-lowering therapy (%)	14.0	5.0	4.0	16.0	0.4	22.0	10.0	33.0	67.0	<0.001
Antihypertensive therapy (%)	16.0	0.0	27.0	83.0	<0.001	18.0	20.0	50.0	83.0	<0.001
Physical activity: never or sporadically (%)	42	50	45.5	63	0.07	45	51	48	68	0.08
Current smokers (%)	26	20	25	31	0.68	15	15	27	20	0.02

The data were expressed as follows: ^a^means (±SD) or ^b^medians (25th and 75th percentiles); em dash means no data; ^!^*p* < 0.001, ^&^*p* < 0.01, ∗*p* < 0.05; 1a versus 2a or 3a versus 4a or 1b versus 2b or 3b versus 4b, all differences are adjusted for WC with the exception of variables (age, BMI, WC, and years after menopause) and percentage variables; ^#^*p* < 0.001, ^$^*p* < 0.01, ^@^*p* < 0.05; 1a versus 1b, 2a versus 2b, 3a versus 3b, 4a versus 4b. NFG: normal fasting glucose; IFG: impaired fasting glucose.

**Table 3:** Association of FSH, LH, and LH/FSH ratio with metabolic disturbances.

*Progression from NFG to IFG*	FSH^1^	FSH^2^	FSH^3^	FSH^4^	FSH^5^	FSH^6^	FSH^7^	FSH^8^	FSH^9^
OR (95% CI) per 1 SD decrease in *z*-score of FSH	2.83^&^ (1.3 - 6.0)	2.88^&^ (1.4 - 6.1)	2.91^&^ (1.2 - 7.0)	2.3∗ (1.0 - 4.7)	2.1∗ (0.9 - 3.8)	2.9^&^ (1.4 - 6.3)	2.59^&^ (1.2 – 5.6)	2.49∗ (1.1 – 5.3)	2.82^&^ (1.3 – 5.9)
*Progression from IFG to diabetes*	FSH^1^	FSH^2^	FSH^3^	FSH^4^	FSH^5^	FSH^6^	FSH^7^	FSH^8^	FSH^9^
OR (95% CI) per 1 SD decrease in *z*-score of FSH	8.49^&^ (4.6 -18.0)	8.64^&^ (3.0 - 20.0)	5.27^&^ (2.8 - 10.0)	4.3^&^ (2.1 - 9.5)	6.2^&^ (2.8 - 15.1)	7.5^&^ (3.4 - 17.2)	8.2^&^ (4.3 –17.3)	8.70^&^ (5.1 –19.3)	8.59^&^ (3.2 –19.6)
*Progression to HOMA-IR* > 2.0	FSH^1^	FSH^2^	FSH^3^	FSH^4^	FSH^5^	FSH^6^	FSH^7^	FSH^8^	FSH^9^
OR (95% CI) per 1 SD decrease in *z*-score of FSH	5.0^!^ (2.3 - 10.7)	5.2^!^ (2.4 - 11.4)	4.7^!^ (2.0 - 11.1)	4.4^&^ (1.9 - 10.3)	4.34^&^ (1.9 - 9.9)	4.3^&^ (1.9 - 10.0)	4.49^&^ (2.1 – 9.7)	4.47^&^ (1.8 –11.3)	4.78^!^ (2.3 –10.1)
*Progression from NFG to IFG*	LH^1^	LH^2^	LH^3^	LH^4^	LH^5^	LH^6^	LH^7^	LH^8^	LH^9^
OR (95% CI) per 1 SD decrease in *z*-score of LH	1.14 (0.7 - 2.0)	1.09 (0.6 - 2.0)	0.96 (0.5 -1.7)	1.07 (0.6 - 1.9)	1.17 (0.6 - 2.2)	1.15 (0.7 – 2.0)	1.0 (0.6 – 1.6)	1.11 (0.4 – 3.0)	1.22 (0.7 – 2.2)
*Progression from IFG to diabetes*	LH^1^	LH^2^	LH^3^	LH^4^	LH^5^	LH^6^	LH^7^	LH^8^	LH^9^
OR (95% CI) per 1 SD decrease in *z*-score of LH	3.5∗ (1.0 - 11.9)	3.54∗ (1.0 - 12.0)	1.15 (0.21 - 6.0)	2.8 (0.6 –12.0)	4.17∗ (1.0 -13.0)	3.1∗ (0.9 - 10.0)	3.6∗ (1.0 –12.7)	3.1∗ (0.8 –10.0)	3.60∗ (1.0 –12.8)
*Progression to HOMA-IR* > 2.0	LH^1^	LH^2^	LH^3^	LH^4^	LH^5^	LH^6^	LH^7^	LH^8^	LH^9^
OR (95% CI) per 1 SD decrease in *z*-score of LH	2.6^&^ (1.4 - 4.7)	2.51^&^ (1.4 - 4.6)	2.05∗ (1.1 - 3.8)	2.2∗ (1.2 - 4.1)	2.2^&^ (1.2 - 4.1)	2.45^&^ (1.2 - 5.0)	2.56^&^ (1.3 – 5.0)	2.17∗ (1.1 – 4.4)	2.69! (1.5 – 4.8)
*Progression from NFG to IFG*	LH/FSH^1^	LH/FSH^2^	LH/FSH^3^	LH/FSH^4^	LH/FSH^5^	LH/FSH^6^	LH/FSH^7^	LH/FSH^8^	LH/FSH^9^
OR (95% CI) per 1 SD increase in *z*-score of LH/FSH	2.03∗ (1.1 - 3.7)	2.05∗ (1.1 - 3,7)	1.96∗ (1.1 - 3.5)	1.95∗ (1.1 - 3.5)	1.38 (0.7 - 2.9)	2.1∗ (1.1 – 3.8)	1.96∗ (1.1 – 3.6)	1.94∗ (0.8 – 3.1)	1.98∗ (1.1 – 3.6)
*Progression from IFG to diabetes*	LH/FSH^1^	LH/FSH^2^	LH/FSH^3^	LH/FSH^4^	LH/FSH^5^	LH/FSH^6^	LH/FSH^7^	LH/FSH^8^	LH/FSH^9^
OR (95% CI) per 1 SD increase in *z*-score of LH/FSH	1.16 (0.6 - 2.2)	1.25 (0.6 - 2.5)	1.64 (0.6 - 3.8)	1.7 (0.7 - 4.2)	0.93 (0.4 - 2.0)	1.15 (0.6 – 2.2)	1.2 (0.6 – 2.3)	1.45 (0.6 – 3.5)	1.17 (0.6 – 2.2)
*Progression to HOMA-IR* > 2.0	LH/FSH^1^	LH/FSH^2^	LH/FSH^3^	LH/FSH^4^	LH/FSH^5^	LH/FSH^6^	LH/FSH^7^	LH/FSH^8^	LH/FSH^9^
OR (95% CI) per 1 SD increase in *z*-score of LH/FSH	1.55 (1.0 - 2.5)	1.39 (0.9 - 2.2)	1.39 (0.8 - 2.3)	1.4 (0.9 - 2.4)	1.33 (0.8 - 2.3)	1.31 (0.7 - 2.3)	1.38 (0.8 – 2.3)	1.58 (0.9 – 2.7)	1.50 (0.9 – 2.4)

Model 1 crude model; model 2 crude model adjusted for age; model 3 crude model adjusted for WC; model 4 crude model adjusted for BMI; model 5 crude model adjusted for CRP; model 6 crude model adjusted for TG; model 7 crude model adjusted for HDL; model 8 crude model adjusted for systolic blood pressure; model 9 crude model adjusted for 17beta-estradiol. *p* level of Wald statistic,^!^*p* < 0.001, ^&^*p* < 0.01, ∗*p* < 0.05 for all models achieved that statistical goodness-of-fit according to log-likelihood statistics.

## Data Availability

No data were used to support this study.
